# Main Factors Determining the Economic Production Sustained by Public Long-Term Care Spending in Spain

**DOI:** 10.3390/ijerph18179199

**Published:** 2021-08-31

**Authors:** Fernando Bermejo, Raúl del Pozo, Pablo Moya

**Affiliations:** School of Social Sciences, Castilla-La Mancha University (UCLM), 16071 Cuenca, Spain; Raul.delPozo@uclm.es (R.d.P.); Pablo.Moya@uclm.es (P.M.)

**Keywords:** long-term care, public spending, economic crisis, structural analysis

## Abstract

Policy reforms of 2012 introduced in Spain a set of austerity measures to emerge from the 2008 global recession. However, attaining the sustainability of the long-term care (LTC) system by reducing public spending overlooks the drawbacks of a lower demand to meet dependency needs. In this context, this study is intended to provide a deeper insight into the economic factors affecting the shifts in the industrial output sustained by LTC spending before and after the austerity measures adopted in 2012. To accomplish this, we first apply a model based on the Input-Output methodology to quantify the output arisen from the consumption demand to meet the dependency needs covered by LTC spending in 2009, 2012 and 2015. Using the results of this model, we carry out a Structural Decomposition Analysis to explore the main drivers of change in the Spanish economic production for 2009–2012 and 2012–2015. The findings reveal that LTC demand factors have proven more relevant than technology factors in increasing production for the two periods considered. Such findings might guide political decision-making on the management of the LTC system in Spain, showing that public LTC spending does not merely contribute to the welfare of dependents, but also may boost economic production.

## 1. Introduction

### 1.1. Background and Review of the Literature

The demand for long-term care (LTC, onwards) services is becoming increasingly relevant in Spain, where 2.6% of the total population is dependent (1.2 million individuals according to the Economic and Social Council of Spain [1]); seven out of every ten dependents are over the age of 65, and more than half are beyond the age of 80 [2]. To face this challenge, Act 39/2006 of 14th December on Promotion of Personal Autonomy and Assistance for Persons in a Situation of Dependency [3] (known as the Dependency Act, henceforth DA) was passed in 2006. The DA began to be applied during the most outstanding prosperity phase of the Spanish economy, but soon experienced the budgetary constraints imposed by the outbreak of the 2008 global financial crisis. Thus, the fiscal consolidation program implemented by 2010 introduced minor amendments in the LTC system affecting the retroactivity of benefits and the assessment periods [4]. Nevertheless, the persistence of severe fiscal imbalances forced the implementation of two structural reforms in 2012 [5,6]. The following measures were introduced: the maximum amount allocated to cash benefits by the DA was reduced; the intensities of service provision decreased; the effective start-up of allowances for potential beneficiaries with moderate or lesser dependency levels was postponed for three years. In addition, the assignment of the co-payment to be faced by dependents rose up to funding more than 50% of the LTC costs. Such measures were aligned with the mainstream positions holding that larger public spending would cause a budgetary deficit that should be assumed by the Administration, which would curb economic growth by increasing public debt beyond a reasonable limit [7]. While introducing this new regulation on co-payment was intended to reduce public deficit [8,9], it has subsequently proven to cause a significant impoverishing effect, increasing the probability of impoverishment in the population affected by 18.9% [10].

However, the adoption of previous measures ignores essential aspects as regards the adequacy of the Spanish LTC system. According to Stiglitz [11], the expenditure on social protection schemes during economic downswings should automatically go up, helping to stabilize the economy. In a broad sense, if governments cut social protection plans, the flow of income to beneficiaries decreases, and so does the stabilizing effects of their spending [12]. For the case of this study, cutbacks in the LTC system in Spain would reveal the adverse consequences that a lower demand to meet the dependency needs may cause on the production level of the Spanish economy. Given that LTC spending is very much concentrated in health and social work activities, its contribution should not be merely assessed by the undeniable welfare benefits that it produces, but also because the demand to meet dependency needs helps to boost local and inter-industry production, sustaining jobs and creating income in the Spanish economy.

In light of the above, it seems necessary to gain more insight into the factors affecting LTC spending and its potential economic return. This becomes particularly relevant in a period of major transition from a sharp cutback in social benefits due to the economic collapse of 2009 to the system stabilization in 2015 through the strengthening of measures adopted in 2012.

The literature on LTC public spending is mostly focused on financing aspects or on future spending projections [13,14,15,16]. We also found studies reporting the impact of the LTC system on job creation nationwide [17] and at the Spanish regional level [18]. The LTC system in Spain initially reported a macroeconomic impact of 262,735 new jobs for an economic scenario in 2010, 190,000 induced jobs and the potential incorporation of 115,000 workers from the informal care sector [19]. These results were contrasted by two studies. The first one focused on the economic effect of the National System of Dependence implementation and its capacity for job creation [20]. This study estimated the impact caused by current spending and investment in construction, considering both public initiative and 50% public/private initiative. The results were obtained by using the Input-Output (IO) framework (174,464 jobs), the Hermin model (169,855 jobs) and microeconomic methodology (160,314 jobs). The second study reduced the employment forecasts to 154,523 jobs by also using IO analysis and the Hermin model [21] with an annual average of 137,000 jobs between 2007 and 2011, which led to a fiscal return of 27% via taxes and payroll contributions.

As previously mentioned, the 2008 financial crisis caused two structural reforms that were implemented in 2012, introducing austerity measures that affected the LTC system. Recent studies have analyzed how those modifications impacted production derived from LTC spending in the Spanish economy in terms of job creation [22], considering fiscal return [23], and from an industry-wide view [24] by using IO methodology. Initially developed by Leontief as a tool in interindustry analysis [25], IO models allow researchers to analyze the effects produced in the industrial activity by exogenous changes of final demand and by the exchanges of commodities between economic sectors. The advantage of using these models in quantifying shifts in economic production and capturing spillover effects in the economy makes them a suitable tool for economic impact evaluation in different fields of research as environmental analysis [26,27,28,29,30,31,32], income distribution effects [33,34,35,36,37], Tourism activities [38,39,40,41,42], socio-economic and demographic changes [43,44,45,46,47], household consumption [48,49,50,51,52] or health systems [53,54]. Furthermore, IO models entail the preliminary methodology to apply Structural Decomposition Analysis (SDA). This technique has been extensively used to explore the main factors responsible for changes in the variable of interest [55,56,57,58,59,60,61,62,63,64,65]. No study, however, has been conducted using SDA on LTC spending.

### 1.2. Purpose of the Research

This study aims at contributing to the debate about the effectiveness of health and social work activities in two fundamental ways: (i) by providing empirical evidence of the positive effect that the LTC financed by public funds causes on the economic production in Spain; and (ii) by identifying the main economic factors determining the evolution of the production arisen from LTC. With this purpose, we assess the production arisen from the consumption demand to meet the dependency needs that are funded with LTC spending by using a model based on IO methodology for years 2009, 2012 and 2015. Furthermore, we explore the main drivers of change in the level of production resulting from this IO model to gain a better understanding of the economic impact of LTC spending during these periods. To accomplish this, a SDA has been applied for 2009–2012 and 2012–2015.

To our knowledge, this is the first study using the SDA methodology to shed light on the main drivers of the increase in production derived from public LTC spending. Our results contribute to understanding the impact of LTC public spending on the Spanish economic structure after the global financial crisis. In considering specific years 2009, 2012 and 2015, we analyze the effect of the changes in the LTC system derived from the reforms of 2012, when the global economy was estimated to start recovering from the 2008 financial crisis.

This article is organized as follows: after this Section where the background, review of the literature and purpose of the investigation have been introduced, Section 2 offers the main aspects of the IO model applied, SDA techniques and data sources. Next, Section 3 presents the results obtained from the DEIO model and the SDA, while Section 4 deals with the discussion, and Section 5 concludes.

## 2. Materials and Methods

### 2.1. Modelling the Impact of Public LTC Spending on the Spanish Economy: DEIO Analysis

Our assessment of the economic return on the consumption demand sustained by LTC spending grounds on the IO framework is as follows. Basically, we have used the Dependency Extended Input-Output (DEIO) model built in [24] upon the first approach in [22] and [23], which explicitly consider the consumption by employed and unemployed households as an independent component of final demand. The potential of IO models relies on their ability to quantify shifts in production due to demand shocks, as well as to account for the consumption linkages between the industries of an economy. Extended IO models widen these capabilities by including both income and consumption transactions for different population groups. Hence, the DEIO model proposed here entails a most suitable tool to address the first issue raised in this study, which is quantifying the gain in output caused by the demand sustained by LTC spending when the population groups of employed, unemployed and LTC beneficiaries are explicitly considered.

**Figure 1:**
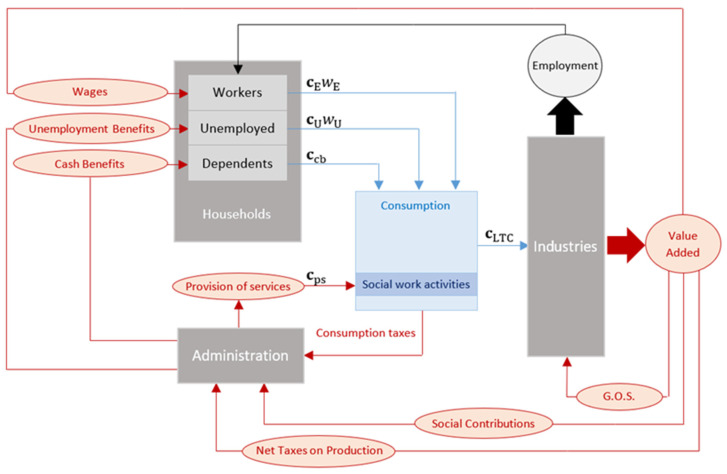
Modeling the socio-economic return on public LTC spending [24].

Figure 1 shows the relevant variables and linkages of the DEIO model, which is further described in Appendix A. In short, the DEIO model is applied to the consumption $c_{\mathrm{LTC}}$ funded with the public spending on LTC for the specific years 2009, 2012 and 2015. Thus, consumption $c_{\mathrm{LTC}}$ entails the demand shock triggering the multiplier effect on output $x_{\mathrm{LTC}}$ that is captured by our DEIO model. In describing this effect, it is useful to remark that the allocation of public spending to the LTC system for consumption $c_{\mathrm{LTC}}$ is directly influenced by the two types of dependency benefits:

- Almost 66% of $c_{\mathrm{LTC}}$ is directly allocated by the Administration for the provision of services $c_{\mathrm{ps}}$, which generates a straightforward increase in consumption in the social work sector
- The remaining 33% of $c_{\mathrm{LTC}}$ is granted via cash benefits $c_{\mathrm{cb}}$, which means the Administration provides the eligible households with an amount of money as payment of informal care. While these sorts of transfers are provided to meet the dependency needs addressed by the LTC system, the ultimate effect is an increase in the total income of dependent households. Assuming that informal care is issued within the family circle [8], no third party payment is made for this service. Then, such a rise in income will effectively ease the budget constraint of households, allowing them to spend a more considerable amount of money not only on social work activities but also on the rest of goods and services.

Based on the DEIO model described in Figure 1, an increase in consumption $c_{\mathrm{LTC}}$ not only causes a proportional gain in gross output (direct impact), but it is also transmitted to the production system where inter-industrial backward linkages generate a multiplicative effect (indirect impact). Such a multiplicative effect arises from the need of all industrial sectors to be provided with the inputs required in their productive processes. Besides industrial inputs, the economic sectors also demand the additional labor required in the production process resulting from the direct and indirect impacts above described, increasing the total wage in the economy. This rise in income is consequently spent by the employed households, generating a subsequent round of consumption shocks (induced impact $c_{E}w_{E}$). Such an induced impact due to wages is further reinforced with the household consumption that is funded via unemployment benefits ($c_{U}w_{U}$). Assuming a fixed labor force, increased demand for workers implies decreasing unemployment, which leads to a change in induced consumption due to the substitution effect between employed and unemployed households.

Following traditional IO techniques, the equilibrium output solution for the DEIO model is determined as follows:

(1) $$x_{\mathrm{LTC}}={\left(I-A-\left[c_{E}w_{E}-c_{U}w_{U}\right]\;{\hat{l}}_{d}\right)}^{-1}\cdot c_{\mathrm{LTC}}$$

where:

- I is the identity matrix
- $A=\left\{a_{ij}\right\}$ is the technical coefficient matrix entailing the consumption of commodity i by economic activity j
- $c_{E}w_{E}$ is the monetary consumption by the employed, which results from applying the consumption propensities $c_{E}$ for the households where the reference person is employed on wages $w_{E}$ arisen from industrial production
- $c_{U}w_{U}$ is the monetary consumption by the unemployed, which results from applying the consumption propensities $c_{U}$ for the households where the reference person is unemployed on unemployment benefit $w_{U}$ paid out by the Government
- $\;{\hat{l}}_{d}$ is a diagonal matrix obtained from the vector of direct labor coefficients $l_{d}=\frac{l_{j}}{X_{j}},$ (being **l** employment and **x** total output by industry **j**).

### 2.2. The Main Drivers of Change in the Output Sustained by Public LTC Spending: SDA Approach

Since the impact of public LTC spending during the evolution of the financial crisis has turned to be relevant, we consider it crucial to explore the underlying factors driving the generation of gross output $x_{\mathrm{LTC}}$ obtained from Equation (1). Thus, the results from the DEIO model above are the inputs for the SDA to be undertaken.

SDA is traditionally used to break down changes in one dependent variable into the changes in its determinants [66]. SDA enables analyses of economic change through a set of comparative static changes in key variables within an IO framework. SDA techniques allow researchers to explore the critical driving forces of change affecting the observed trend of a variable over time [65]. In light of this, SDA is the most suitable tool to address the main issue raised in this study, which is exploring the main drivers of shifts in the production sustained by LTC spending over 2009–2012 and 2012–2015.

**Figure 2:**
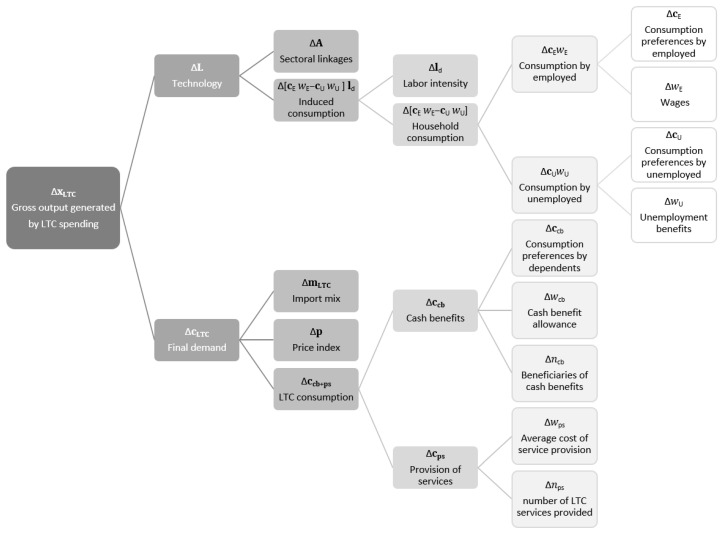
Determinants of gross output generated by LTC spending.

Figure 2 shows the decomposition structure of the determinants explaining changes in $x_{\mathrm{LTC}}$ (further described in Appendix B), which can be firstly decomposed into the effect caused by technology changes and the effect due to changes in final demand $c_{\mathrm{LTC}}$. In the DEIO model, changes in technology refer to those involving the factors included in the extended Leontief inverse (interindustry linkages and the induced consumption by households). In a second level of decomposition of technology, the analysis digs up the effects due to changes in sectoral links as well as in labor coefficients, unemployment benefits, wages, and consumption profiles of both employed and unemployed. As for the second level of decomposition in demand $c_{\mathrm{LTC}}$, the analysis focuses on changes due to the import mix in LTC consumption, prices, and those factors affecting both cash-benefit demand (consumption profile of dependent households, cash-benefit allowance, and number of beneficiaries) and in-kind services provision (average cost of services and number of services provided). Appendix B includes the mathematical aspects on how the factors corresponding to this structural decomposition are estimated.

### 2.3. Data Sources for the DEIO Model and SDA

Data about public spending on LTC split into cash benefits and in-kind services have been recently detailed in the report published by the Commission where the Spanish LTC System [67] was analyzed. As mentioned above, cash benefits and in-kind services (LTC spending) entail the inputs of our DEIO model. The 2009, 2012 and 2015 Symmetric Input-Output Tables (SIOT) at current prices for Spain were extracted from the WIOD Database’s 2016 Release [68,69]. The SIOT is defined at producer prices and adheres to the 2008 version of the System of National Accounts. The information is broken down into 56 sectors, according to the International Standard Industrial Classification (ISIC revision 4). For this investigation, the category of “Health services and social work activities” was split into two major divisions, “Health services” and “Social work activities”, so the final sectoring scheme to be applied in our DEIO model includes 57 industries.

The profiles of consumption for dependent, employed, and unemployed households were collected from the Spanish Household Budget Survey (HBS) [70]. This database provides information on the amount (in purchasing prices) and structure of household expenditures according to the Classification of Individual Consumption by Purpose (COICOP). The HBS also contains socioeconomic data about the standard of living, income, and the professional activity of the household reference person. For the sake of simplicity, we shall assume that a household is classified according to the status of its reference person. By defining specific data filters based on the primary source of income over the HBS sample of roughly 24,000 households, we can determine the distribution of the consumption basket for households receiving LTC benefits. Moreover, the endogenous consumption by employed and unemployed households is also obtained from the HBS by identifying the households where the reference person is employed or unemployed.

Both the consumption funded with spending on LTC and the endogenous consumption of households must be incorporated into the DEIO framework according to the 57-sector scheme previously described. Two data transformations need to be done: (i) Given that expenditure data are measured at purchasing prices in the HBS, consumption for dependent, employed and unemployed households need to be converted to producer prices by removing trade and transportation margins, and indirect taxes to products (including value-added taxes among others); (ii) it is necessary to match HBS microdata referring to commodity expenditures to the industrial sectoring scheme adopted by the SIOT in order to provide comparable results between consumption demand and sectoral production. Since there is no official bridge between the COICOP used in the HBS and the ISIC revision 4 used in the SIOT, a bridging matrix provided by the ONS [71] able to match household consumption with industries has been applied.

A crucial last feature involves the imports incorporated into household consumption. Imported products cause no productive impact in terms of value added and job generation and were therefore excluded. Considering that the HBS does not provide explicit information about expenditures related to imports, the distinction between domestic and imported products is based upon the corresponding distribution of domestic and imported products for the household consumption in the SIOT.

Finally, the vector of sectoral employment to obtain $\;l_{d}$ was extracted from the National Accounting of the Spanish Statistics Institute [72].

## 3. Results

In this section, the aggregated and sector-based findings obtained from the DEIO model previously described are presented. We initially show the results for the consumption and output generated from LTC spending for 2009, 2012, 2015 as well as the differences between those years. Then, the SDA outcomes will shed some light on the main drivers of change in gross output derived from LTC spending during the recession period.

### 3.1. Empirical Results of the DEIO Model

First, Table 1 presents the estimation of LTC spending for the existing types of benefit during the years considered. We observed that LTC spending experienced an increase of 12.6% during the period preceding the implementation of the austerity measures (2009–2012), while the increase was 8% in the subsequent period (2012–2015). Table 1 also includes the number of beneficiaries and the average amount of money allocated to provision of services and cash benefits. These data reflect that both the beneficiaries of provision of services and cash benefits were equally distributed in 2009. Moreover, although the number of beneficiaries of provision of services increased by 11.18% in 2012, the share of beneficiaries of provision of services over the total number of beneficiaries reduced to 39.16% to increase up to approximately 46% further of the total beneficiaries in 2015. Regarding monetary benefits, we observe that the average value of the provision of services is approximately three times the amount allocated to cash benefits for all years. In addition, we observed a significant decrease in the value of the provision of services between 2009 and 2012 (−13.8%) together with a moderate decrease from 2012 to 2015 (−2.5%), while cash benefits experienced a slightly continuous increase by 1% in 2009–2012 and 2% in 2012–2015.

In a second stage, Table 2 shows the monetary values of consumption $c_{\mathrm{LTC}}$ (in basic prices) funded with public LTC spending at constant prices of 2012. This entails the exogenous shock in demand, the effects of which are assessed by the DEIO model are here applied.

As described in the data and methods section, vector $c_{\mathrm{LTC}}\;$ accounts for the consumption by dependent households funded with the informal care benefits $c_{\mathrm{cb}}$ (increasing the whole basket of consumption) plus provision of services $c_{\mathrm{ps}}$ directly paid for by the Administration. We can observe that consumption $c_{\mathrm{LTC}}$ is essentially focused on the Social work activities sector, which intensely determines the structure of production resulting from the model. In addition, we observe important differences in consumption gains, both in the total value, which may be because government spending on the System has reached a saturation point, and in its distribution by sectors, which may be influenced by the implementation of the austerity measures affecting the LTC system. As regards “Social work activities” consumption, we first observe a slight drop of 55.4 M€ between 2009 and 2012 due to the above-mentioned implementation of austerity measures, to experience a significant increase of 760 M€ subsequently during 2012–2015.

Additionally, Table 2 shows the output $x_{\mathrm{LTC}}$ generated by the consumption demand financed with LTC spending for 2009, Δ2009−2012 and Δ2012−2015, reflecting that the total level of production has more than doubled the initial LTC spending for all the years considered. These results prove the multiplier effect described in the DEIO model, but the Social work activities sector has not been particularly beneficial of the effect, specifically in 2012.

### 3.2. SDA Results

Table 3 shows the results of the SDA that has been performed to identify the main determinants driving Δ$x_{\mathrm{LTC}}$ in 2009–2012 and 2012–2015. We find interesting differences between the results for the first and the second period. The gain in gross output rose by 49% between the first and last cross section of our analysis (2009–2015), although this increase was more significant during the period preceding the austerity measures (2009–2012: 30.44%) than the increase during the second period considered (2012–2015: 18.58%). The factors related to LTC demand (share of imports in LTC consumption, price index and LTC domestic) have been more influential than those related to technology (Inter-industry linkages and induced consumption) for the two periods considered. Thus, LTC demand determinants were responsible for almost 60% of the total gross output growth during the first period, while this percentage slightly reduced to 55.4% from 2012 to 2015.

Among the factors explaining changes in LTC demand, the number of beneficiaries of cash benefits proved the most decisive during the first period, while it turned out to be the number of beneficiaries of provision of services during the second period. Moreover, it is also important to note that the number of beneficiaries of cash benefits came to have a negative impact in the second period (−3.34%). As Table 3 shows, changes in the average of monetary value allocated for provision of services would have led to an output drop ceteris paribus of 13.67% during the first period and 4.25% during the second period.

Regarding technology, it should be noted that induced consumption was the most determining factor explaining the increase in production. While changes in consumption by employed hardly contributed to output gains (1.16% in both periods), the variation in consumption by unemployed caused the production to grow by 17.47% (9.85% from 2009 to 2012 plus 7.61% between 2012 and 2015), particularly led by changes related to the unemployment benefit.

## 4. Discussion

To the best of our knowledge, this is the first study focusing on the effects that LTC spending caused in the Spanish economy during the financial crisis by using SDA and IO methodology. The SDA here applied allows researchers to assess the main factors driving the gain in production derived from a changing level in LTC spending between 2009 and 2015. Our analysis reveals that such factors have been decisively influenced by the austerity measures introduced in 2012, which entailed a lower increase in LTC spending from 2012 to 2015 that subsequently led to a smaller gain in production. According to the results obtained, changes in the number of beneficiaries of cash benefits was the most determining factor during the period preceding the implementation of the austerity measures of social policy, while changes in the beneficiaries of provision of services turned to be the most decisive in the second period considered.

The 2008 financial crisis has brought about devastating effects in Spain. GDP dropped by 9% during the period 2008–2013 [73], and the unemployment rate increased from 8% in 2008 to 26% in 2012 [74]. The most important measures taken by the legislator were: changes in taxation and provision of benefits, changes in spending on public services and structural fiscal reforms (fiscal policy and structural public spending reforms) [75]. Among the structural spending reforms, one of the most remarkable measures referred to a drastic structural reform of the DA [5,6], which is still in the implementation period, to reduce the fiscal deficit of public accounts and fulfil fiscal consolidation targets [76,77]. Together with the measures previously explained, the worst consequence of cutbacks in the LTC system was the growth in waiting lists to receive the benefit [78]. In this regard, it is worth mentioning that two out of ten people died without having received the corresponding dependency benefit in 2018 [79]. The effect of the above measures is reflected in the results of our analysis. Thus, the growth rate of the LTC system needed to meet the targets originally conceived about the coverage rate was reduced by 40% as a consequence of the reforms.

In light of the above, our study provides evidence on how the aforementioned changes have affected the Spanish economy. The results reveal that almost 60% of the increase in production was due to LTC demand factors for the period 2009–2012, while the same LTC demand factors were responsible for 55.4% of the increase in production from 2012 to 2015. As for the technology factors, the induced consumption proves more significant than the inter-industry linkages to explain changes in production, the public funds allocated to unemployment benefits being the most determining factor. This is explained by the specific behavior of unemployment in the Spanish economy, which is highly sensitive to economic downturns and recoveries. Thus, due to the devastating effect of the crisis, the unemployment rate increased from 8% in 2008 up to 26% in 2012 [74], while in economic terms, GDP contracted by 9% during the period 2008–2013 [73]. Furthermore, induced consumption by the unemployed has been boosted during the crisis by the allocation of cash benefits instead of provision of services. Following [80], the unemployed households with dependents have chosen cash benefits to supplement unemployment benefits in order to enhance their disposable income and therefore consumption.

Regarding changes in LTC demand, we find two relevant factors. The first one is the number of beneficiaries of cash benefits, which initially caused a positive impact in the 2009–2012 period to become further negative in the second period (2012–2015). Such a trend could be explained by the fact that cash benefits involve a lower cost for all local administrations and a shorter waiting time for dependents, so this type of benefits has been overused instead of being applied on an exceptional basis [3,21,81]. After the 2012 structural reform [5,6], the criteria for granting cash benefits have hardened, which has resulted in a reduction in the number of informal caregivers in favor of the number of people hired to support the provision of services [21,82].

The second factor is related to the determinants explaining the consumption of provision of services (the average amount of money allocated to service provision and the number of beneficiaries). First, decreasing the average amount of money allocated to provision of services generates a negative impact on production, although considerably lower in the second period than in the first one. Such a decrease is essentially based on two grounds: (i) the insufficient development of the service network in Spain and the fragility of the fundamentals on which the system was built. Basically, we must consider the weakness of the initial financing design of the LTC system, which has hardly begun to improve in the second period under analysis [79,80]. In addition, (ii) the variation in the service mix offered to the beneficiaries during the periods of time considered is of note. Thus, both residential and institutional care had more relative weighting compared to home care services and day centers for the elderly during 2009–2012, while residential services were reduced to boost home care services for 2012–2015, and, considering absolute terms, the provision of services increased to replace cash benefits.

Secondly, regarding the number of beneficiaries, it shows the symmetrical but opposite behavior of cash benefits: hardening the conditions for assigning them meant a transfer of users to provision of services [83].

Lastly, some limitations and advantages of the DEIO model need to be mentioned. The main contribution of IO modelling grounds on its ability to quantify not only the direct impacts on production that are caused by an exogenous shock in demand, but also the spillover effects on the local economy. Thus, the DEIO model here applied is able to provide results including the gross output generated by the demand arisen from LTC spending plus both the indirect impacts (those arising from inter-industry connections) and induced impacts (those arising from income-consumption connections) on production. However, a remarkable constraint of IO basic models is its exogenous treatment of unemployment by assuming that the only income-earners in an economy are the employed. As already highlighted in a seminal paper by Batey and Weeks [45], if the specific demand by the unemployed is ignored when production levels change, the induced effect obtained in basic IO models can be overestimated. To avoid such bias, the DEIO model has been built taking into consideration demographic variables, following the structure of the so-called demo-economic models [84] able to manage consumption responses by different population groups (employed and unemployed in the specific case of the present analysis) which supposes an important improvement in the precision of our results.

## 5. Conclusions

While increasing public spending on social protection helps achieve the objectives of sustainability and adequacy of the LTC system, it is deemed to curtail economic growth potentially by crowding out private investment and other public expenditures. This seems to have been the main argument behind the austerity measures introduced in Spain in 2012 to limit public spending on social protection. However, the approach of attaining the sustainability of the LTC system by reducing public spending overlooks the adverse consequences that a lower demand in LTC services may cause on the economy. Nevertheless, while spending is an important constraint, policymakers should bear in mind the unquestionable contributions of the LTC system, not only to social welfare but also as a source of additional demand able to boost economic growth. 

Given the widespread concern upon public spending on LTC, the present study contributes to the debate about the effectiveness of health and social work activities in two fundamental ways: (i) by providing empirical evidence of the positive effect that the LTC financed by public funds causes on the Spanish economy in generating production; and (ii) by identifying the main economic determinants driving the evolution of the production arisen from LTC during the financial crisis. With this purpose, we have applied a SDA to explore the main drivers of change in the output sustained by public spending on LTC before and after the austerity measures adopted in 2012.

The results of the SDA reveal that LTC demand factors have proven more relevant than technology in increasing production for the two periods considered. Thus, LTC demand determinants were responsible for almost 60% of the total gross output growth during the first period, while this percentage slightly reduced to 55.4% from 2012 to 2015. The number of beneficiaries of cash benefits was the main factor explaining changes in LTC demand before the adoption of the austerity measures, while the number of beneficiaries of provision of services turned to be the most decisive factor after 2012. The results also show that the output arisen from the LTS system has been highly sensitive to changes in the induced consumption by households, particularly the unemployed.

To conclude, the findings obtained from both the DEIO model and the SDA might guide political decision-making on the management of the LTC system in Spain. Such results may also be useful to provide more insight into the crucial role that the LTC system plays for the Spanish society, not only in terms of social welfare but also from the economic perspective. However, a particular limitation of this study needs to be mentioned. The results are very specific and cannot be generalized to other economies because each country has its own system and population structure. In any case, the DEIO model proposed here, as well as the SDA techniques applied, are widely applicable to any other country where IO tables and consumption data required as inputs were available. Thus, extending this analysis to other economies may undoubtedly be regarded as a future line of research.

## Figures and Tables

**Table 1:** Estimation of LTC spending by type of benefit.

	LTC Spending (Million Euros)	Provision of Services			Cash Benefits		
		*n*_ps_^1^	*%n*_ps_^2^	*w*_ps_^3^ (euros)	*n*_cb_^4^	*%n*_cb_^5^	*w*_cb_^6^ (Euros)
2009	6124.03	285,185	49.87%	15,901.20	286,723	50.13%	5124.00
2012	6895.00	317,063	39.16%	13,700.27	492,622	60.84%	5178.72
2015	7449.00	380,592	45.95%	13,357.99	447,679	54.05%	5282.93

^1^ *n*_ps_ Beneficiaries of provision of services. ^2^ *n*_ps_ Percentage of beneficiaries of provision of services over total beneficiaries. ^3^ *w*_ps_ Average of monetary value allocated for provision of services. ^4^ *n*_cb_ Beneficiaries of cash benefits. ^5^ *n*_ps_ Percentage of beneficiaries of cash benefits over total beneficiaries. ^6^ *w*_cb_ Average of monetary value allocated for cash benefits.

**Table 2:** Consumption and total output generated by LTC spending.

	Social Work Activities Sector		Total (All Sectors)	
	Million Euros	Percentual Change	Million Euros	Percentual Change
Consumption ^1^ $c_{\mathrm{LTC}}$**:**				
Base year (2009)	4319.1		5465.82	
Δ2009−2012	−55.3	−1.28	922.88	16.88
Δ2012−2015	704.7	16.53	513.68	8.04
Output ^2^ $x_{\mathrm{LTC}}$:				
Base year (2009)	4394.9		13,573.6	
Δ2009−2012	−46.6	−1.06	4131.86	30.44
Δ2012−2015	720.1	16.56	2521.91	14.24

^1^ Consumption and Output in basic prices. Million euros of 2012. ^2^ Consumption and Output for Social work activities sector represents a 79.02% and 32.38% of the total sectors, respectively.

**Table 3:** SDA of the output $x_{\mathrm{LTC}}$ generated by LTC spending.

	2009–2012		2012–2015	
	Million € of 2012	(%)	Million € of 2012	(%)
A. TECHNOLOGY factors	1670.63	12.31	1125.46	8.29
1. Inter-industry linkages	357.40	2.63	−39.16	−0.29
2. Induced consumption	1420.60	10.47	1107.52	8.16
2.1. Induced consumption by employed	83.43	0.61	73.92	0.54
2.1.1. Consumption coefficients of employed households	114.31	0.84	−14.28	−0.11
2.1.2. Wages	−30.87	−0.23	88.19	0.65
2.2. Induced consumption by unemployed	1337.17	9.85	1033.60	7.61
2.2.1. Consumption coefficients of unemployed households	−23.36	−0.17	36.84	0.27
2.2.2. Unemployment benefits	1360.53	10.02	996.77	7.34
2.3. Labor intensity	−107.38	−0.79	57.09	0.42
B. LTC DEMAND factors	2461.23	18.13	1396.45	10.29
1. Share of imports in LTC consumption	−89.14	−0.66	−32.14	−0.24
2. Price index	670.16	4.94	13.03	0.10
3. LTC domestic consumption at constant prices of 2012	1880.21	13.85	1415.56	10.43
3.1. Consumption derived from cash benefits	2534.46	18.67	−469.10	−3.46
3.1.1. Distribution of consumption funded with cash benefits	76.21	0.56	−68.17	−0.50
3.1.2. Average of monetary value allocated for cash benefits	203.03	1.50	52.54	0.39
3.1.3. Beneficiaries of cash benefits	2255.22	16.61	−453.47	−3.34
3.2. Consumption derived from provision of services	−654.25	−4.82	1884.66	13.88
3.2.1. Average of monetary value allocated for provision of services	−1855.68	−13.67	−576.63	−4.25
3.2.2. Beneficiaries of provision of services	1201.44	8.85	2461.29	18.13
Total output	4131.86	30.44	2521.91	18.58

## Data Availability

The authors used datasets in this study that are publicly available. These data can be found here: http://www.wiod.org/database/wiots16 for WIOD data, https://www.ine.es/dyngs/INEbase/es/operacion.htm?c=Estadistica_C&cid=1254736177056&menu=resultados&idp=1254735576581 for employment data and https://www.ine.es/dyngs/INEbase/es/operacion.htm?c=Estadistica_C&cid=1254736176806&menu=resultados&idp=1254735976608#!tabs-1254736194790 for HBS data.

## Appendix A. The Dependency Extended Input-Output (DEIO)

Following the demo-economic model described by Batey [84], our DEIO model first separates consumption by employed and unemployed households from the rest of final demand to transfer both subsequently to the inter-industry transaction matrix, so they behave as any other industrial activity. By doing so, an increase in industrial output will also increase induced consumption, which in turn will be reflected in higher levels of inter-industry consumption. The equilibrium of the DEIO model can be expressed in matrix form as follows:

(A1) $$\left(\begin{array}{ccc} I-A & -c_{E}w_{E} & -c_{U}w_{U}\\ -l_{d} & 1 & 0\\ 0 & 1 & 1 \end{array}\right)\cdot \left(\begin{array}{c} x\\ e\\ u \end{array}\right)=\left(\begin{array}{c} c_{\mathrm{LTC}}+f_{R}\\ s\\ p \end{array}\right)$$

where:

- The first block on the left-hand side refers to an extended matrix of intermediate demand with the following components:
  ○ the traditional Leontief matrix I−A where the technical coefficient matrix $A=\left\{a_{ij}\right\}$ represents the consumption of commodity i by economic activity j that is subtracted from the identity matrix **I**
  ○ the (column) vector $c_{E}w_{E}$ of monetary consumption by the employed, which results from applying the consumption propensities $c_{E}$ for the households where the reference person is employed on wages $w_{E}$ arisen from industrial production
  ○ the (column) vector $c_{U}w_{U}$ of monetary consumption by the unemployed, which results from applying the consumption propensities $c_{U}$ for the households where the reference person is unemployed on unemployment benefit $w_{U}$ paid out by the Government
  ○ the (row) vector of direct labor coefficients $l_{d}$ representing the number of jobs required per unit of production by industry
- The second block on the left-hand side is the vector (column) of activity levels containing industrial gross output **x**, employed *e* and unemployed *u*.
- The block on the right-hand side is the vector (column) of inputs including final demand $\left\{c_{\mathrm{LTC}}+f_{R}\right\}$, external level of employment *s* = 0 and total labor force *p*. As previously said, $c_{\mathrm{LTC}}$ denotes the consumption demand funded with LTC spending, while $f_{R}$ contains the remaining final demand excluding both the employed and unemployed consumption previously transferred to intermediate demand.

The model can also be presented as three simultaneous equations:

(A2) $$\left(I-A\right)x-c_{E}w_{E}\;e-c_{U}w_{U}\;u=c_{\mathrm{LTC}}+f_{R}$$

(A3) $$-l_{d}x+e=0$$

(A4) e+u=p

As Equation (A2) shows, the extended intermediate demand $Ax-c_{E}w_{E}\;e-c_{U}w_{U}\;u$ comprises not only the inter-industry consumption of commodity **Ax** in the traditional Leontief matrix but also the induced consumption of employed and unemployed ($c_{E}w_{E}e$ and $c_{U}w_{U}\;u$, respectively) that is endogenously determined depending on the solution of the three simultaneous equations above-defined. Such a solution involves Equation (A3) where the gross output requirements of employment $l_{d}x$ are translated into labor demand *e*, and Equation (A4) where the demographic balance is explicitly introduced by defining labor supply *p* equal to labor demand *e* plus unemployment *u*. As noted in [36], Equation (A4) reflects the reduction (or increase) in unemployed resulting from an increase (or decrease) in industrial production.

All the above considered, the system has the following closed-form solution for total output:

(A5) $$x={\left(I-A-\left[c_{E}w_{E}-c_{U}w_{U}\right]\;l_{d}\right)}^{-1}\cdot \left\{c_{\mathrm{LTC}}+f_{R}+c_{U}w_{U}\;p\right\}$$

The effect that allocating public LTC spending may cause on total production $x_{\mathrm{LTC}}$ in a given year is estimated in two steps: firstly, we obtain a baseline result on production **x** by shocking the economy with the full final demand. Secondly, the production $x_{0}$ generated by the final demand excluding the exogenous consumption derived from LTC spending $c_{\mathrm{LTC}}$ is estimated. Then, $x_{0}$ is subtracted from the previous baseline result **x**. Therefore:

(A6) $$x_{\mathrm{LTC}}=x-x_{0}={\left(I-A-\left[c_{E}w_{E}-c_{U}w_{U}\right]\;l_{d}\right)}^{-1}\cdot c_{\mathrm{LTC}}$$

that is Equation (1) stated in previous Section 2.1 on Materials and Methods about modelling the impact of public LTC spending on the Spanish economy.

## Appendix B. Structural Decomposition Analysis for Output $x_{LTC}$

As previously mentioned, SDA is traditionally used to break down changes in one dependent variable into the changes in its determinants, which are assumed to be independent. Initially formulated by Skolka [85], SDA is still widely used to analyze economic impact evaluation with respect to output, value added, employment and other socioeconomic indicators. Following [86], changes in the gross output depending on an exogenous demand shock can be decomposed into two main effects: (1) an indirect effect caused by technology changes in the extended Leontief inverse; and (2) the effect due to changes in the final demand under consideration.

In the present study where $x_{\mathrm{LTC}}={\left(I-A-\left[c_{E}w_{E}-c_{U}w_{U}\right]\;l_{d}\right)}^{-1}\cdot c_{\mathrm{LTC}}$, if extended Leontief inverse ${\left(I-A-\left[c_{E}w_{E}-c_{U}w_{U}\right]\;l_{d}\right)}^{-1}$ is called L, that means decomposing the total change in output $x_{\mathrm{LTC}}=L\cdot c_{\mathrm{LTC}}$ into its driving factors will be as follows:

(A7) $$\Delta x_{\mathrm{LTC}}=\Delta L\;c_{\mathrm{LTC}}+L\;\Delta c_{\mathrm{LTC}}+\Delta L\;\Delta c_{\mathrm{LTC}}$$

The solution of an additive SDA as in the above-proposed is not unique. As explained by Rose and Casler [87], Dietzenbacher and Los [65] and Arto and Dietzenbacher [88], the solution depends on the size of the residuals arisen from the interaction among the considered determinants. Consequently, according to the technique described in [65], the interactions among the determinants **L** and $c_{\mathrm{LTC}}$ have been hereby addressed by taking the average of the two polar decompositions and using midpoint weights:

(A8) $$\Delta x_{\mathrm{LTC}}=\left(1/2\right)\Delta L\;\left(c_{\mathrm{LTC}}{}^{0}+c_{\mathrm{LTC}}{}^{1}\right)+\left(1/2\right)\left(L^{0}+L^{1}\right)\;\Delta c_{\mathrm{LTC}}$$

The next sections describe the structural decomposition approach to examine the causes of change in determinants **L** and $c_{\mathrm{LTC}}$.

### Appendix B.1. Decomposition of Changes in Technology (L)

Data about public spending on LTC split into cash benefits and in-kind services have been recently detailed in the report published by the Commission where the Spanish Long-Term. For this investigation, the category of “Health services and social work activities” was split into two major divisions, “Health services” and “Social work activities”, so the final sectoring scheme to be applied in our DEIO model includes 57 industries.

As presented in Equation (A6), the extended Leontief inverse **L** is composed of both industrial linkages A and consumption interactions $\left[c_{E}w_{E}-c_{U}w_{U}\right]\;l_{d}$ that need to be isolated. Based on [39], this inverse matrix can be decomposed into a sum of partitions of the extended direct input requirement matrix as $\Delta L=L^{1}\Delta A_{D}L^{0}$, where $A_{D}=A+\left[c_{E}w_{E}-c_{U}w_{U}\right]\;l_{d}$. Hence, ΔL is decomposed into six components:

(A9) $$\Delta L=L^{1}\Delta AL^{0}+L^{1}\Delta A^{\mathrm{Ec}}L^{0}+L^{1}\Delta A^{\mathrm{Ew}}L^{0}-L^{1}\Delta A^{\mathrm{Uc}}L^{0}-L^{1}\Delta A^{\mathrm{Uw}}L^{0}+L^{1}\Delta A^{\mathrm{ld}}L^{0}$$

Table A1 describes the six components in Equation (A9) and how they are estimated.

**Table A1:** Definition of components involving the SDA of changes in technology (L).

Decomposition of changes in technology (L)	$\Delta A=A^{1}-A^{0}$	changes in the local sectoral linkages
	$\Delta A^{\mathrm{Ec}}=\left(1/2\right)\Delta c_{E}\left(w_{E}^{1}l_{d}^{1}+w_{E}^{0}l_{d}^{0}\right)$	changes in the consumption preferences of households where the reference person is employed
	$\Delta A^{\mathrm{Ew}}=\left(1/2\right)\left(c_{E}^{0}\left(\Delta w_{E}\right)l_{d}^{1}+c_{E}^{1}\left(\Delta w_{E}\right)l_{d}^{0}\right)$	changes in annual wages
	$\Delta A^{\mathrm{Uc}}=\left(1/2\right)\Delta c_{U}\left(w_{U}^{1}l_{d}^{1}+w_{U}^{0}l_{d}^{0}\right)$	changes in the consumption preferences of households where the reference person is unemployed
	$\Delta A^{\mathrm{Uw}}=\left(1/2\right)\left(c_{U}^{0}\left(\Delta w_{U}\right)l_{d}^{1}+c_{U}^{1}\left(\Delta w_{U}\right)l_{d}^{0}\right)$	changes in unemployment benefits
	$\Delta A^{\mathrm{ld}}=\left(1/2\right)\left(\left(c_{E}^{1}w_{E}^{1}-c_{U}^{1}w_{U}^{1}\right)+\left(c_{E}^{0}w_{E}^{0}-c_{U}^{0}w_{U}^{0}\right)\right)\Delta l_{d}$	changes in the labor demand coefficients

### Appendix B.2. Decomposition of Changes in LTC Demand ($c_{LTC}$)

According to the SDA technique described in [64,89], changes in final demand related to consumption are driven by two main effects: the product mix effect and the final demand level effect. Regarding our specific model, final demand entails the consumption sustained by LTC spending $c_{LTC}$ including, as mentioned in the section om Materials and Methods, the consumption by the beneficiaries of cash benefits $c_{\mathrm{cb}}$ and the consumption related to the provision of services $c_{\mathrm{ps}}$. Changes in $c_{\mathrm{cb}}$ can be further explained by changes in the consumption profile of dependent households and by changes in the level of consumption derived from variations in both benefits $w_{\mathrm{cb}}$ and beneficiaries $n_{cb}$. As regards $c_{\mathrm{ps}}$, changes will be explained just by changes in the level of consumption due to the amount of money allocated to social work services $w_{ps}\;$ and the number of dependents $n_{ps}$ covered with those services.

Both $c_{\mathrm{ps}}$ and $c_{\mathrm{cb}}$ need to be adjusted from purchasing values at current prices to domestic values at constant prices. First, LTC consumption profiles should refer to goods and services locally produced since imports do not cause a direct impact on gross domestic product nor local job creation. Secondly, exploring the variation in consumption in real terms will allow us to add the inflation effect to the drivers of change in the production arisen from LTC spending. Since the study provides results for changes in production through three moments in time (2009, 2012 and 2015), adjusting consumption from current prices to real prices by using the price index will allow us to prove whether the variation in gross output for the time periods 2009–2012 and 2012–2015 has been due to the volume of industrial production or to price rises.

All the above considered, LTC consumption can be defined as follows:

(A10) $$c_{\mathrm{LTC}}=m_{\mathrm{LTC}}\;p_{2012}\;c_{\mathrm{cb}+\mathrm{ps}}=m_{\mathrm{LTC}}\;p_{2012}\;(c_{\mathrm{cb}}\;w_{cb}\;n_{cb}+w_{ps}\;n_{ps})$$

while the variation in $c_{\mathrm{LTC}}$ can be described by using the following SDA method:

(A11) $$\Delta c_{\mathrm{LTC}}=\Delta c_{\mathrm{LTC}}{}^{m}+\Delta c_{\mathrm{LTC}}{}^{p}+\Delta c_{\mathrm{cb}}{}^{c}+\Delta c_{\mathrm{cb}}{}^{w}+\Delta c_{\mathrm{cb}}{}^{n}+\Delta c_{\mathrm{ps}}{}^{w}+\Delta c_{\mathrm{ps}}{}^{n}$$

Table A2 describes the components in Equation (A11) and how they are estimated.

**Table A2:** Definition of components involving the SDA of changes in LTC demand ($c_{LTC}$).

Decomposition of changes in LTC demand ($c_{LTC}$)	$\Delta c_{\mathrm{LTC}}{}^{m}=\left(1/2\right)\Delta m_{\mathrm{LTC}}\left(p_{\mathrm{LTC}}^{1}\;c_{\mathrm{cb}+\mathrm{ps}}^{1}+p_{\mathrm{LTC}}^{0}\;c_{\mathrm{cb}+\mathrm{ps}}^{0}\right)$	changes in the import shares of LTC consumption
	$\Delta c_{\mathrm{LTC}}{}^{p}=\left(1/2\right)\left(m_{\mathrm{LTC}}^{0}\;\Delta p_{\mathrm{LTC}}\;c_{\mathrm{cb}+\mathrm{ps}}^{1}+m_{\mathrm{LTC}}^{1}\;\Delta p_{\mathrm{LTC}}\;c_{\mathrm{cb}+\mathrm{ps}}^{0}\right)$	changes in LTC consumption due to inflation
	$\Delta c_{\mathrm{cb}}{}^{c}=\left(1/2\right)\left(m_{\mathrm{LTC}}^{1}\;p_{\mathrm{LTC}}^{1}+m_{\mathrm{LTC}}^{0}\;p_{\mathrm{LTC}}^{0}\right)\left[\left(1/2\right)\Delta c_{\mathrm{cb}}\left(w_{\mathrm{cb}}^{1}\;n_{\mathrm{cb}}^{1}+w_{\mathrm{cb}}^{0}\;n_{\mathrm{cb}}^{0}\right)\right]$	changes in the distribution of cash-benefit consumption (product-mix effect on LTC consumption)
	$\Delta c_{\mathrm{cb}}{}^{w}=\left(1/2\right)\left(m_{\mathrm{LTC}}^{1}\;p_{\mathrm{LTC}}^{1}+m_{\mathrm{LTC}}^{0}\;p_{\mathrm{LTC}}^{0}\right)\left[\left(1/2\right)c_{\mathrm{cb}}^{0}\left(\Delta w_{\mathrm{cb}}\right)n_{\mathrm{cb}}^{1}+c_{\mathrm{cb}}^{1}\left(\Delta w_{\mathrm{cb}}\right)n_{\mathrm{cb}}^{0}\right]$	changes in cash-benefit allowances (level effect on demand via cash benefits)
	$\Delta c_{\mathrm{cb}}{}^{n}=\left(1/2\right)\left(m_{\mathrm{LTC}}^{1}\;p_{\mathrm{LTC}}^{1}+m_{\mathrm{LTC}}^{0}\;p_{\mathrm{LTC}}^{0}\right)\left[\left(1/2\right)\left(c_{\mathrm{cb}}^{1}w_{\mathrm{cb}}^{1}+c_{\mathrm{cb}}^{0}w_{\mathrm{cb}}^{0}\right)\Delta n_{\mathrm{cb}}\right]$	changes in the number of cash-benefit receivers (level effect on demand via cash-benefit beneficiaries)
	$\Delta c_{\mathrm{ps}}{}^{w}=\left(1/2\right)\left(m_{\mathrm{LTC}}^{1}\;p_{\mathrm{LTC}}^{1}+m_{\mathrm{LTC}}^{0}\;p_{\mathrm{LTC}}^{0}\right)\left[\left(1/2\right)\Delta w_{\mathrm{ps}}(n_{\mathrm{ps}}^{1}+n_{\mathrm{ps}}^{0})\right]$	changes in the average cost of the LTC service provision (level effect on demand via provision of services)
	$\Delta c_{\mathrm{ps}}{}^{n}=\left(1/2\right)\left(m_{\mathrm{LTC}}^{1}\;p_{\mathrm{LTC}}^{1}+m_{\mathrm{LTC}}^{0}\;p_{\mathrm{LTC}}^{0}\right)\left[\left(1/2\right)\left(w_{\mathrm{ps}}^{1}+w_{\mathrm{ps}}^{0}\right)\Delta n_{\mathrm{ps}}\right]$	changes in the number of LTC services provided (level effect on demand via service-provision beneficiaries)
